# Metabolomic and Microbiome Profiling Reveals the Protective Mechanism of *Pyrrosia petiolosa* Against Radiation-Induced Intestinal Injury

**DOI:** 10.3390/ijms27125279

**Published:** 2026-06-10

**Authors:** Hua Yang, Hansheng Zhu, Xin Yan, Yimeng Liu, Yiping Chen, Jia Wang, Jian Zhang, Min Huang, Mianxue Liu, Hao Shi, Yue Zhou, Changyi Huang, Zhihui Zhang, Shiying Yan, Jian Zhao, Qian Chen

**Affiliations:** 1 Key Laboratory of Biological Resource and Ecological Environment of Chinese Education Ministry, College of Life Sciences, Sichuan University, Chengdu 610064, China; 17807062156@163.com (H.Y.);; 2 Irradiation Preservation and Effect Key Laboratory of Sichuan Province, Sichuan Institute of Atomic Energy, Chengdu 610101, China

**Keywords:** radiation-induced intestinal injury, *Pyrrosia petiolosa* (Christ) Ching, gut microbiota, untargeted metabolomics, microbiome–metabolome integration

## Abstract

Radiation-induced intestinal injury (RIII) is a common complication of tumor radiotherapy, significantly impacting patients’ quality of life and posing challenges for developing effective medical countermeasures. This study investigated the reparative effects of the traditional Chinese medicine *Pyrrosia petiolosa* (Christ) Ching on radiation damage through in vivo and in vitro models. By integrating gut microbiota and untargeted metabolomics analyses, it elucidated the multidimensional mechanisms through which *P. petiolosa* regulates the microbiome as well as metabolic homeostasis. In vitro experiments demonstrated that *P. petiolosa* effectively suppressed radiation-induced inflammatory factors (*IL-6*, *TNF-α*, and *IL-1β*) and alleviated radiation-induced oxidative stress (MDA, GSH, and SOD). In vivo models further confirmed that *P. petiolosa* significantly alleviated radiation-induced intestinal inflammation and leukopenia, while protecting the structural and functional integrity of mouse small intestinal crypt villi. Mechanistic studies revealed *P. petiolosa* reshaped the gut microbiota by promoting enrichment of beneficial bacteria such as Bacteroides, concurrently restoring the homeostasis of key metabolic pathways, including glutathione, glycerophospholipids, and the tricarboxylic acid cycle. Analysis of the microbiome–metabolome interaction network revealed that treatment with *P. petiolosa* altered the correlation patterns between gut microbiota and fecal metabolites, including potentially beneficial bacteria and metabolites associated with inflammatory and oxidative stress responses. These findings suggest that microbiome–metabolome remodeling may contribute to the protective effects of *P. petiolosa* against radiation-induced intestinal damage. Overall, this study provides preliminary evidence that *P. petiolosa* may alleviate acute radiation-induced intestinal damage through anti-inflammatory and antioxidant effects accompanied by changes in gut microbiota and metabolic homeostasis, while identifying candidate targets for future functional validation.

## 1. Introduction

During abdominal or pelvic radiotherapy, the intestine is particularly vulnerable to radiation-induced intestinal injury (RIII), owing to its marked radiosensitivity. This condition can compromise the integrity of the intestinal mucosal barrier [1], leading to acute gastrointestinal syndrome and, in severe cases, life-threatening complications [2]. Currently, over 50% of cancer patients undergo radiotherapy as part of their standard treatment regimen [3]. However, approximately 75% of these patients develop radiation-induced intestinal complications of varying severity [4]. Even more concerning, nearly 90% of patients receiving pelvic radiotherapy experience permanent alterations in bowel habits, and about 50% report a significant decline in quality of life following treatment [5]. RIII is characterized by inflammation and tissue damage in radiosensitive regions of the gastrointestinal tract caused by ionizing radiation exposure [6]. It is commonly classified as acute or chronic according to the timing of symptom onset. Acute RIII usually occurs during or within a few weeks following radiotherapy, presenting with symptoms such as diarrhea, abdominal pain, and mucosal ulceration [7]. In contrast, chronic RIII may occur months to decades after treatment and can lead to serious complications, including intestinal obstruction, fibrosis, and perforation, with consequent deterioration in quality of life and therapeutic outcomes [8]. Current clinical interventions for RIII, including pharmacological therapy [9], probiotics [10], and surgical procedures, have shown limited efficacy and often fail to achieve satisfactory outcomes. Consequently, the development of novel therapeutic strategies to alleviate RIII has emerged as a critical focus in the fields of radiation medicine and gastrointestinal research.

*Pyrrosia petiolosa* (Christ) Ching, an epiphytic fern belonging to the *Pyrrosia genus* in the family Polypodiaceae [11], is a widely distributed medicinal plant that is rich in bioactive compounds such as eugenol, kaempferol, quercetin, and iso-mangiferin [12]. Modern pharmacological research has revealed that *P. petiolosa* possesses a broad spectrum of bioactivities [13], including anti-inflammatory, antioxidant, immunomodulatory, antibacterial, diuretic, and wound-healing effects [14]. Recent studies have demonstrated that intragastric administration of an aqueous extract of *P. petiolosa* at various concentrations significantly attenuates inflammatory infiltration, enhances SOD activity, and reduces nitric oxide levels in rats with ammonium oxalate-induced nephrolithiasis [15]. Ethyl acetate extract of *P. petiolosa* inhibited the growth of Gram-negative bacteria [16]. *P. petiolosa* powder and its active components, quercetin and kaempferol, reduced urinary oxalate levels by modulating oxalate metabolism, thereby alleviating renal tissue damage and preventing the formation of kidney stones [17].

Despite its well-documented pharmacological properties, research on the therapeutic potential of *P. petiolosa* in RIII remains scarce. Interestingly, its multitarget effects—including anti-inflammatory, antioxidant, and microbiota-modulating activities—are highly aligned with the complex pathophysiological features of RIII [2]. Therefore, this study combines cellular experiments, animal models, gut microbiome analysis, and metabolomics to systematically evaluate the reparative effects of *P. petiolosa* on RIII and to explore its potential mechanisms, with the aim of providing experimental evidence for the use of natural products in the prevention and treatment of RIII.

## 2. Results

### 2.1. P. petiolosa Reduces Radiation-Induced Inflammatory Expression Levels and Alleviates Cellular Oxidative Stress in MODE-K Cells

**Figure 1:**
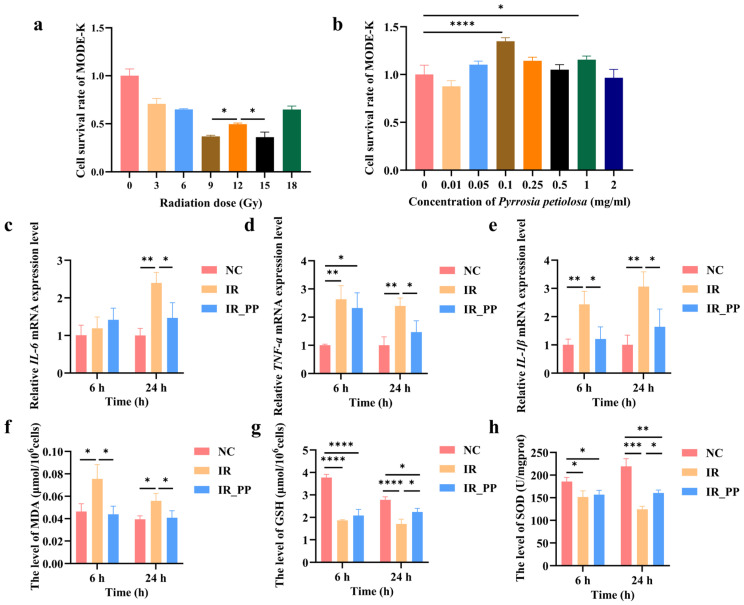
The protective effect of *P. petiolosa* on irradiated MODE-K cells. (**a**) The survival rate of cells 24 h after irradiation was assessed using the CCK-8 assay (*n* = 6). (**b**) The survival rate of cells after 24 h of exposure to different concentrations of *P. petiolosa* (*n* = 6). (**c**) The relative expression levels of *IL-6* at different time points (6 h and 24 h) post-irradiation were detected by qPCR (*n* = 3). (**d**) The relative expression levels of *TNF-α* at different time points (6 h and 24 h) post-irradiation were detected by qPCR (*n* = 3). (**e**) The relative expression levels of *IL-1β* at different time points (6 h and 24 h) post-irradiation were detected by qPCR (*n* = 3). (**f**) The level of MDA was measured at different time points (6 h and 24 h) following irradiation (*n* = 3). (**g**) The level of GSH was measured at different time points (6 h and 24 h) following irradiation (*n* = 3). (**h**) The level of SOD was measured at different time points (6 h and 24 h) following irradiation (*n* = 3). Each experiment was performed in triplicate, and data are presented as mean ± SD. * *p* < 0.05, ** *p* < 0.01, *** *p* < 0.001, and **** *p* < 0.0001.

To establish a cellular irradiation injury model, MODE-K cells were first subjected to X-ray irradiation at varying doses (0–18 Gy) (Figure 1a). The results showed that overall cell survival declined in a dose-dependent manner with an increasing radiation dose. Compared to non-irradiated cells, survival rates were significantly lower in all irradiated groups (*p* < 0.05), dropping to 30% at 15 Gy, indicating high sensitivity of MODE-K cells to ionizing radiation. A radiation dose of 12 Gy, corresponding to 50% cell survival, was selected to construct an in vitro model of radiation-induced intestinal epithelial injury. The dose significantly reduced cell viability, effectively simulating severe radiation damage and facilitating the evaluation of radiation-protective potential. Subsequently, the safety and optimal intervention concentration of *P. petiolosa* were first assessed (Figure 1b). CCK-8 assays showed that *P. petiolosa* significantly enhanced cell viability at both 0.1 mg/mL and 1 mg/mL, indicating a potential pro-survival effect within the dose range, with the most pronounced effect observed at 0.1 mg/mL (*p* < 0.0001). Consequently, all subsequent experiments employed a 0.1 mg/mL concentration.

Then, the effects of *P. petiolosa* on radiation-induced inflammatory responses were evaluated by measuring the mRNA levels of *IL-6*, *TNF-α*, and *IL-1β*. At 6 h post-irradiation, compared with the NC group, the IR group exhibited significantly elevated mRNA expression levels of *TNF-α* (Figure 1d) and *IL-1β* (Figure 1e) (*p* < 0.05), with this elevated expression persisting up to 24 h (*p* < 0.01). At both 6 h and 24 h post-irradiation, expression levels of these two inflammatory factors were significantly suppressed in the IR_PP group treated with *P. petiolosa* (*p* < 0.05 and *p* < 0.01). Although *IL-6* mRNA expression also showed an upward trend in the IR group, intervention with *P. petiolosa* demonstrated a significant inhibitory effect at 24 h (*p* < 0.01) (Figure 1c). Additionally, we measured intracellular oxidative stress levels at different time points as an indicator of cell damage. Compared to the NC group, the IR group exhibited significantly elevated levels of the lipid peroxidation product MDA at both 6 h and 24 h post-irradiation (*p* < 0.05) (Figure 1f). *P. petiolosa* treatment mitigated radiation-induced lipid peroxidation, with a significant reduction in MDA levels (*p* < 0.05). Alterations in the intracellular endogenous antioxidant system were similarly observed and manifested as decreased antioxidant levels post-irradiation. Compared to the NC group, endogenous antioxidant GSH levels sharply decreased at 6 h and 24 h post-irradiation (*p* < 0.0001) and were significantly reversed by *P. petiolosa* at all observed time points (*p* < 0.0001) (Figure 1g). With the depletion of GSH, the level of SOD also significantly decreased (*p* < 0.05), while the *P. petiolosa* intervention significantly restored the level (Figure 1h). The results above indicate that *P. petiolosa* mitigates radiation-induced injury to MODE-K cells by suppressing inflammatory responses and improving oxidative stress conditions.

### 2.2. P. petiolosa Mitigates Radiation-Reduced Intestinal Injury in Mice

**Figure 2:**
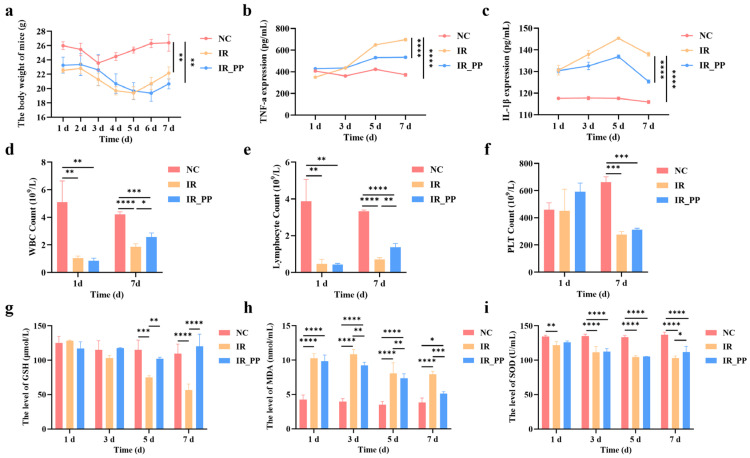
*P. petiolosa* mitigated radiation-induced systemic damage in mice. (**a**) Changes in body weight of mice 7 days after irradiation (*n* = 8). (**b**) TNF-α expression was detected by ELISA at different time points post-irradiation (days 1, 3, 5, and 7) (*n* = 3). (**c**) IL-1β expression was detected by ELISA at different time points post-irradiation (days 1, 3, 5, and 7) (*n* = 3). (**d**–**f**) Blood count (WBC, lymphocyte, PTL) analysis in mice at different time points (days 1 and 7) following irradiation (*n* = 3). (**g**) The level of GSH was measured at different time points (days 1, 3, 5, and 7) following irradiation (*n* = 3). (**h**) The level of MDA was measured at different time points (days 1, 3, 5, and 7) following irradiation (*n* = 3). (**i**) The level of SOD was measured at different time points (days 1, 3, 5, and 7) following irradiation (*n* = 3). * *p* < 0.05, ** *p* < 0.01, *** *p* < 0.001, and **** *p* < 0.0001.

To further investigate the recovery effects of *P. petiolosa* on RIII in mice, we successfully established a model by exposing mice to a dose (7.5 Gy) of radiation. The radiation dose of 7.5 Gy was determined based on our preliminary survival and tolerance optimization experiments. In brief, we evaluated X-ray radiation doses at different levels (0, 2.5, 5, 7.5, and 10 Gy). During the experiments, the 10 Gy dose resulted in complete mortality among the mice, while the 5 Gy dose caused insufficient or mild intestinal damage. Therefore, 7.5 Gy was ultimately selected as the optimal dose to establish a stable, reproducible model of RIII while avoiding excessively high acute mortality rates. The body weight decreased significantly post-irradiation, reaching its lowest point on days 5–6, while the NC group maintained stable weight and gradually increased (Figure 2a). The mice gradually exhibited disheveled fur and diarrhea symptoms on day 3 post-irradiation. By day 6, one mouse in the IR group died, while no deaths occurred in the other groups. Overall, irradiation caused weight loss in mice, accompanied by pronounced clinical symptoms, with a certain recovery trend emerging between days 6 and 7. Simultaneously, radiation induced a systemic inflammatory response. Compared with the NC group, the serum levels of TNF-α (Figure 2b) and IL-1β (Figure 2c) in the IR group significantly increased over time, peaking on days 7 and 5, respectively (*p* < 0.0001). The intervention with *P. petiolosa* significantly suppressed the expression of these proinflammatory factors. On days 5 and 7, both *TNF-α* and IL-1β levels in the IR_PP group were significantly lower than those in the IR group (*p* < 0.001 and *p* < 0.0001).

Blood routine results further revealed the effects of radiation on the hematopoietic system. Compared with the NC group, the IR group exhibited a sharp decline in WBC (Figure 2d) and lymphocyte (Figure 2e) levels on both days 1 and 7 post-irradiation (*p* < 0.01 and *p* < 0.0001), indicating acute bone marrow suppression and impaired immune function. Intervention with *P. petiolosa* significantly ameliorated this condition. At both time points, WBC and lymphocyte levels in the IR_PP group were significantly higher than those in the IR group (*p* < 0.001 and *p* < 0.0001). Additionally, the PLT level (Figure 2f) showed minimal change on day 1 post-irradiation but decreased significantly on day 7 (*p* < 0.001). Irradiation induced marked alterations in serum antioxidant and lipid peroxidation levels in mice. The GSH level (Figure 2g) in the IR group gradually decreased and was significantly lower than that of the NC group after day 5 (*p* < 0.001). MDA levels (Figure 2h) increased on day 1 and remained elevated, indicating enhanced lipid peroxidation. SOD activity (Figure 2i) also markedly decreased post-irradiation. *P. petiolosa* treatment significantly mitigated radiation-induced lipid peroxidation, with MDA levels in the IR_PP group being consistently lower than those in the IR group at all time points (*p* < 0.01 and *p* < 0.0001). Corresponding to the elevated MDA levels was the depletion of the endogenous antioxidant system, with both GSH and SOD levels in the IR_PP group being significantly higher than those in the IR group (*p* < 0.01 and *p* < 0.0001).

**Figure 3:**
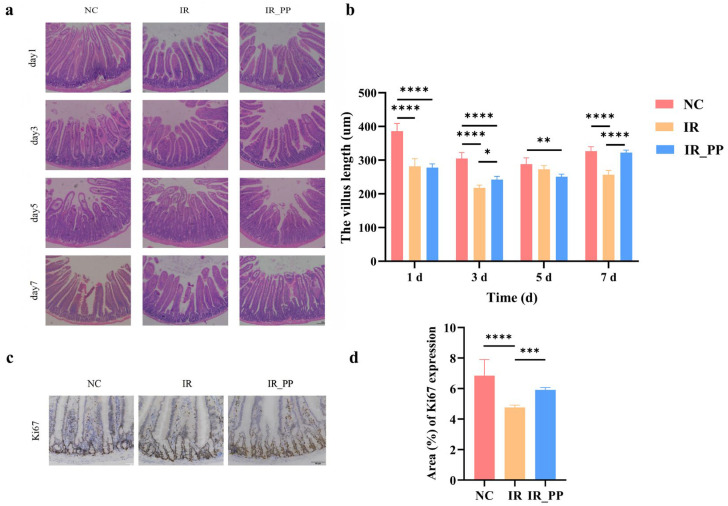
*P. petiolosa* alleviated radiation-induced damage to intestinal tissues. (**a**,**b**) Representative HE staining images of intestinal tissue on days 1, 3, 5, and 7 post-radiation, and the quantification of the length of crypt villi (*n* = 3). (**c**,**d**) Representative images and quantitative analysis of Ki67 IHC staining in intestinal tissue on day 7 post-radiation. Scale bar: (**a**) 100 µm (*n* = 3), (**c**) 50 µm. * *p* < 0.05, ** *p* < 0.01, *** *p* < 0.001, and **** *p* < 0.0001.

HE staining was performed on days 1, 3, 5, and 7 to assess the histology of mouse small intestinal tissue (Figure 3a). The length of the villus was measured as a morphometric indicator of mucosal damage (Figure 3b). Histological images revealed that the NC group exhibited an intact intestinal epithelial structure at all time points, with well-preserved and uniformly distributed villi, neatly arranged crypts, and no significant pathological alterations. In contrast, the IR group exhibited pronounced tissue disruption as early as day 1, characterized by markedly reduced villus height, a sparse and fragmented structure, and shallow crypts. Damage intensified by days 3 and 5, revealing extensive mucosal degeneration and necrosis, indicating entry into the acute phase of RIII. By day 7, although the IR group showed slight structural recovery, it remained below normal levels, suggesting a prolonged recovery period for radiation-induced tissue damage. The IR_PP group consistently demonstrated tissue-protective effects throughout the experiment. Notably, by days 3 and 5, villi exhibited improved alignment and length restoration, accompanied by reduced inflammatory cell infiltration. Quantitative data on the villus length showed high consistency with the histological observations: the villus length in the NC group consistently remained above 400 µm; the IR group exhibited a significant decrease to 250–300 µm, while the IR_PP group demonstrated significantly higher values than the IR group at all time points. Corresponding to the results of Ki67 immunohistochemistry, Ki67-positive cells were widely distributed in the crypts of the small intestine in the NC group (Figure 3c). Following irradiation, Ki67-positive cells in the mouse crypts were significantly reduced. The intervention group treated with *P. petiolosa* exhibited more Ki67-positive cells than the IR group, with the positive area restored to the NC group level (Figure 3d). In summary, the results indicate that *P. petiolosa* provides significant protection against radiation-induced systemic damage in mice by suppressing systemic inflammatory responses, improving oxidative stress conditions, and alleviating damage to the hematopoietic system.

### 2.3. P. petiolosa Modulates Gut Microbiota Composition Following Radiation Exposure

**Figure 4:**
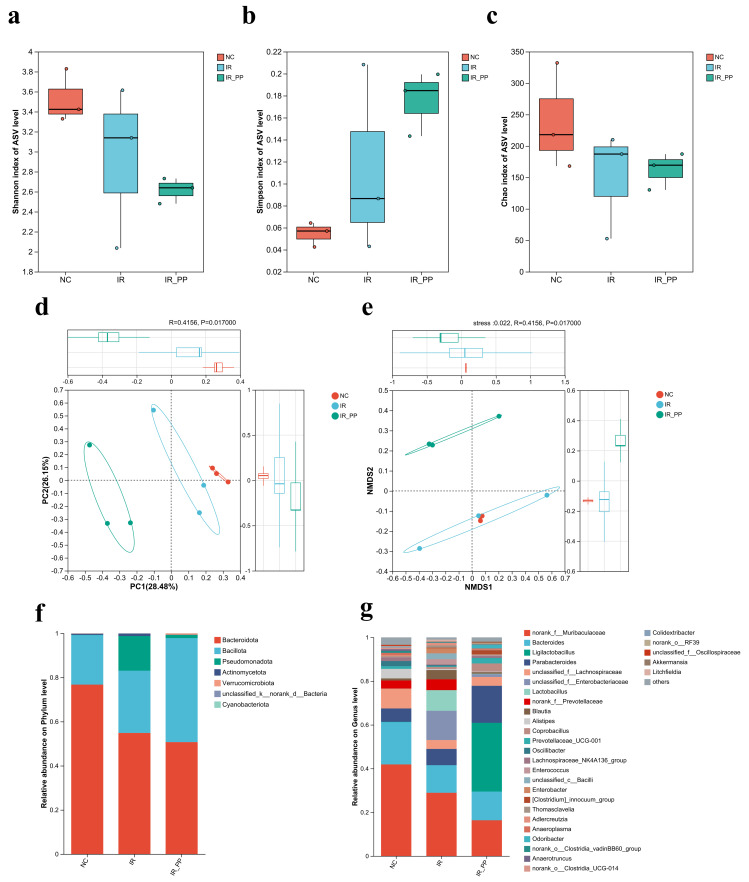

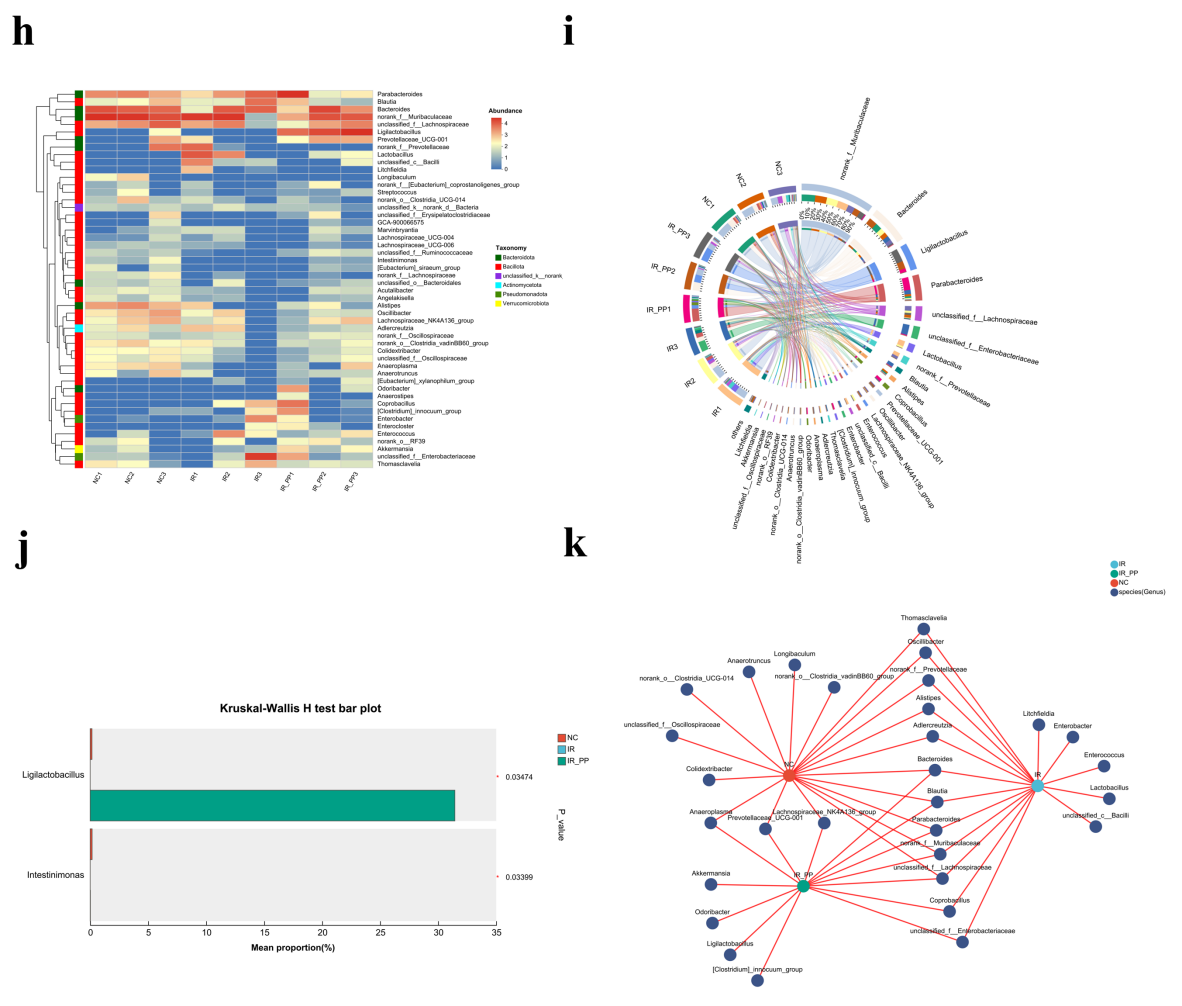
The effect of *P. petiolosa* on the microbial profile of the intestinal flora in mice (*n* = 3). (**a**) Shannon index of the ASV (Amplicon Sequence Variants) level. (**b**) Simpson index of the ASV level. (**c**) Chao index of the ASV level. (**d**) Principal Coordinates Analysis of the genus level. (**e**) Non-metric Multidimensional Scaling of the genus level. (**f**) The relative abundances of microorganisms in the NC group, IR group, and IR_PP group at the phylum level. (**g**) The relative abundances of microorganisms in the NC group, IR group, and IR_PP group at the genus level. (**h**) Heatmap analysis of the relative abundances of bacterial genus among NC, IR, and IR_PP groups. (**i**) Circos analysis of the relative abundances of bacterial genus among NC, IR, and IR_PP groups. (**j**) Analysis of microbiome differences at the genus level. Statistical analysis was performed by the Tukey–Kramer test. (**k**) Network analysis of microbiome differences at the genus level. * *p* < 0.05.

To investigate the regulatory effect of *P. petiolosa* on X-ray-induced intestinal microbiota dysbiosis in mice, microbiome analysis was performed on fecal samples from the three groups of mice using high-throughput sequencing. The alpha diversity of the gut microbiota was assessed using Shannon (Figure 4a), Simpson (Figure 4b), and Chao (Figure 4c) indices. The results indicate that there were no statistically significant differences in the α-diversity indices among the three groups. Nevertheless, certain numerical trends were observed: compared with the NC group, the IR group showed a slight decrease in the Shannon and Chao indices, while the Simpson index increased. This visually suggested a potential downward trend in post-irradiation community richness and diversity, though this lacks statistical support. Interestingly, the Simpson index in the IR_PP group showed a non-significant upward trend, which may tentatively suggest a potential shift in community structure toward enrichment of specific core bacterial genera; however, due to the lack of statistical significance, this observation requires further validation. In contrast, β-diversity analysis (including PCoA and NMDS) revealed significant differences in community structure among the NC, IR, and IR_PP groups (*p* < 0.05), indicating modulation of the gut microbiota characteristics of mice. Samples from the IR_PP group were biased toward the NC group in both PCoA (Figure 4d) and NMDS (Figure 4e) plots, suggesting that *P. petiolosa* intervention may have partially alleviated radiation-induced dysbiosis.

In order to more precisely characterize the distinct changes in each group, the relative abundance of the gut microbiota at the phylum (Figure 4f) and genus levels (Figure 4g) was analyzed. At the phylum level, Bacteroidota and Bacillota (Firmicutes) were predominant in the gut microbiota of the three groups of mice. Compared with the NC group, the relative abundance of Bacteroidota decreased in the IR group, while the abundance of Bacillota increased, accompanied by an expansion of the Pseudomonadota. Following the intervention with *P. petiolosa*, the abundance of Bacteroidota decreased compared to the IR group, the relative abundance of Bacillota increased, and Pseudomonadota also decreased markedly. Additionally, Actinomycetota and Verrucomicrobiota exhibited low abundance across all groups, with minimal variation in abundance. At the genus level, the abundance of dominant bacteria such as *norank_f_Muribaculaceae* decreased in the IR group, while the relative abundance of potential pathogenic bacteria such as *unclassified_f_Enterobacteriaceae* and *Enterococcus* increased. Following the intervention with *P. petiolosa*, the abundance of the probiotic *Ligilactobacillus*, which is believed to play an important role in maintaining intestinal barrier function and anti-inflammatory responses, significantly increased compared to the NC and IR groups (*p* < 0.05).

Circos plots (Figure 4i) and microbial community interaction networks (Figure 4k) further validated the reconstruction of connections between health-related bacterial genera. The NC group exhibited connections with a larger number of dominant bacterial genera, including *norank_f_Muribaculaceae*, *Bacteroides*, *Oscillibacter*, *Alistipes*, and *Prevotellaceae_UCG-001*. Most of these bacteria belong to the gut core microbiota and are involved in polysaccharide decomposition, short-chain fatty acid production, and maintaining intestinal barrier function.

The IR group was closely associated with *unclassified_f_Enterobacteriaceae*, *Enterococcus*, *Enterobacter*, and *Lactobacillus*. Some of these genera are opportunistic pathogens, and their increased abundance may be related to radiation-induced gut microbiota dysbiosis and barrier damage. The IR_PP group exhibited distinct network characteristics, connected to bacteria such as *Ligilactobacillus*, *Akkermansia*, and *Odoribacter*. Among these, *Ligilactobacillus* and *Akkermansia* are probiotics known to enhance intestinal barrier function, regulate host immunity, and exhibit anti-inflammatory effects, suggesting that *P. petiolosa* intervention may promote the colonization and functional recovery of probiotics. Overall, the network analysis reveals that irradiation significantly altered the co-occurrence patterns of the gut microbiota, characterized by abnormal enrichment of potential pathogenic bacteria, while *P. petiolosa* intervention helps to restore a healthy microbial ecological network structure.

### 2.4. Untargeted Metabolomics Reveals Metabolic Reprogramming Induced by P. petiolosa

**Figure 5:**
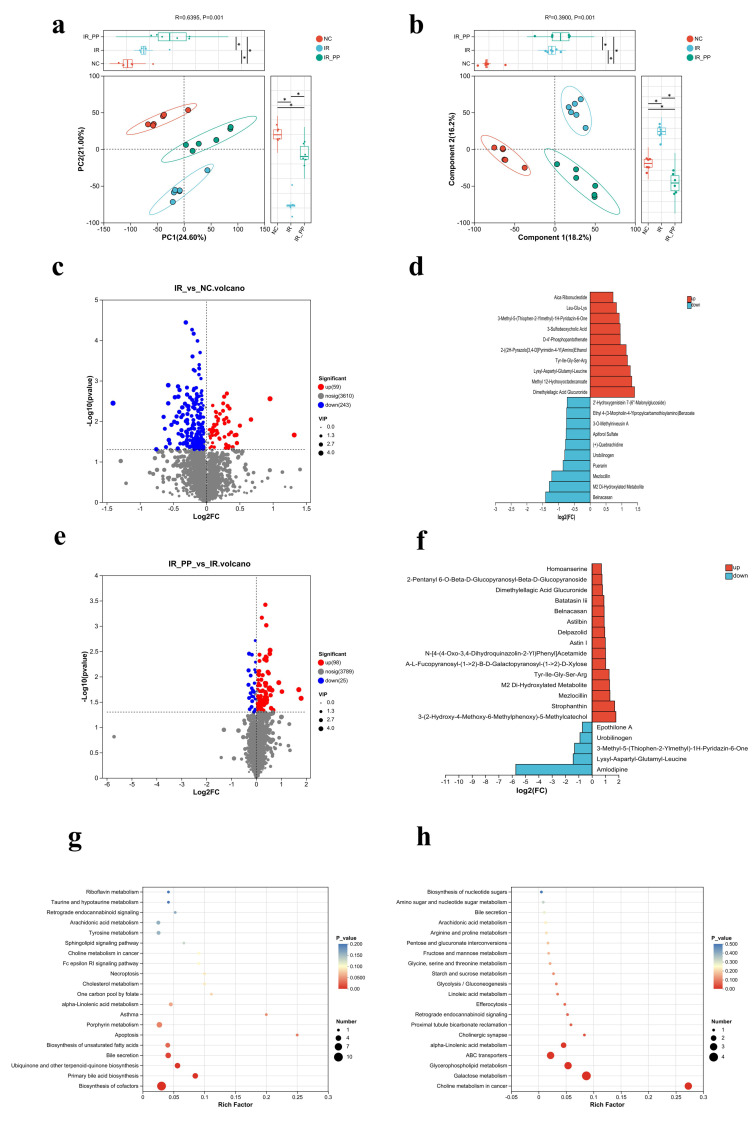
Changes in untargeted metabolomics of mice feces (*n* = 6). (**a**) Principal component analysis (PCA) of all fecal metabolites. (**b**) Partial least squares discriminant analysis (PLS-DA) of all fecal metabolites. (**c**) Volcano plot of the NC and IR groups (vertical dashed line: fold-change = 1; horizontal dashed line: *p* = 0.05). (**d**) The differential metabolites in the top 20 of the NC and IR groups. (**e**) Volcano plot of the IR and IR_PP groups. (vertical dashed line: fold-change = 1; horizontal dashed line: *p* = 0.05). (**f**) The differential metabolites in the top 20 of the IR and IR_PP groups. (**g**) KEGG enrichment analysis of the NC and IR groups. (**h**) KEGG enrichment analysis of the IR and IR_PP groups. * *p* < 0.05.

To investigate the effects of *P. petiolosa* intervention on the gut metabolome of irradiated mice, untargeted metabolomics analysis was performed on fecal samples. PCA (Figure 5a) and PLS-DA (Figure 5b) provided an overall view of the differences between the three groups. In the PCA score plot, the IR group separated from the NC group along PC1, indicating irradiation-associated metabolic divergence. The IR_PP group shifted toward NC on PC1 but formed a distinct cluster along PC2, consistent with a partially restored yet re-equilibrated metabolic state. To illustrate overall metabolite distribution patterns, pairwise comparisons among the three groups were conducted, and volcano plots were generated based on metabolite peak intensities (Figure 5c,e). These plots reveal the important roles played by metabolites under exposure to ionizing radiation and *P. petiolosa* intervention. A total of 598 metabolites were annotated. Compared with the NC group, 302 metabolites were dysregulated in the IR group, including 59 upregulated and 243 downregulated metabolites. Compared with the IR group, 123 metabolites were dysregulated in the IR_PP group, with 98 metabolites upregulated and 25 downregulated.

Next, differential metabolite statistics were performed on the top 20 differentially expressed substances between the two groups (Figure 5d,f). In the NC and IR groups, several amino acids/peptides (Leu-Glu-Lys, Tyr-Ile-Gly-Ser-Arg, and Lysyl-Aspartyl-Glutamyl-Leucine) were significantly elevated. However, at the same time, antioxidant and anti-inflammatory polyphenols/flavonoids (Dimethylellagic Acid Glucuronide and Apiforol sulfate) and endogenous protective substances ((+)-Quebrachidene) were significantly reduced. These results indicate that irradiation induces metabolic changes characterized by stress-related upregulation of amino acids/peptides, while antioxidants, detoxification, and immune homeostasis-related metabolites show widespread decline. Overall, this reflects increased energy demand coupled with diminished protective capacity. In the IR and IR_PP groups, the IR_PP group exhibited significant changes in the metabolic profile. Amino acids and peptides (Homoanserine and Tyr-Ile-Gly-Ser-Arg) were further upregulated, enhancing protein synthesis and tissue repair capacity. The levels of phenols and flavonoids (Astilbin and Dimethylellagic Acid Glucuronide) were significantly restored or increased. These findings suggest that the therapeutic effects of *P. petiolosa* may be associated with the regulation of fecal metabolic profiles, which are linked to nutritional metabolism, oxidative stress, and inflammatory responses. However, since fecal metabolites may originate from the host, gut microbiota, diet, or administered extract, the biological source of these metabolites and their direct functional contributions require further validation. KEGG enrichment analysis revealed that the differentially expressed metabolites between the NC and IR groups were primarily enriched in pathways related to amino acid metabolism, lipid metabolism, bile acid metabolism, and energy metabolism (Figure 5g). In the comparison between IR_PP and IR, enriched pathways included galactose metabolism, glycerophospholipid metabolism, amino acid metabolism, and choline metabolism (Figure 5h). These pathway-level results should be interpreted as functional annotations of the differences in fecal metabolites rather than direct evidence of pathway activation or inhibition. Taken together, these findings suggest that the *P. petiolosa* intervention is associated with a partial remodeling of the fecal metabolome following radiation exposure.

KEGG pathway enrichment analysis revealed that irradiation caused significant disruption of multiple metabolic pathways (Figure 5g), primarily involving cofactor metabolism, bile acid metabolism, porphyrin metabolism, apoptosis, and unsaturated fatty acid biosynthesis pathways. In contrast, the *P. petiolosa* intervention (Figure 5h) (IR vs. IR_PP) significantly enriched pathways such as choline metabolism (cancer-related), α-linolenic acid metabolism, galactose metabolism, and phospholipid metabolism. These results indicate that irradiation primarily causes porphyrin-bile acid metabolic disorders, impaired lipid and cofactor synthesis, and apoptosis activation, while *P. petiolosa* reverses irradiation-induced metabolic disorders by enhancing glucose metabolism and phospholipid metabolism, improving choline metabolism, and maintaining membrane homeostasis, thereby exhibiting a systemic protective effect.

### 2.5. Integrated Microbiome–Metabolome Analysis Uncovers the Mechanistic Basis of P. petiolosa’s Protective Effects

**Figure 6:**
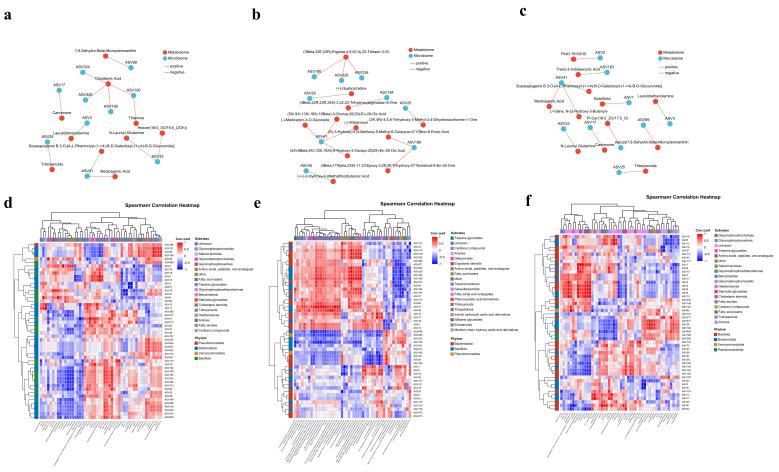
Integrated microbiome–metabolome analysis reveals potential connections between gut microbiota and host metabolism. (**a**–**c**) Microbiome–metabolome correlation network based on Spearman’s rank correlation coefficients. Each node represents one genus (bule) or metabolite (red); two nodes are linked if the correlation coefficient value is > 0.6 and *p* (FDR-corrected) is < 0.001. (**d**–**f**) Heatmap analysis of microbiome–metabolome correlations based on Spearman’s rank correlation coefficients. The top 100 microorganisms and metabolites were selected for analysis. (**a**,**d**) The NC group, (**b**,**e**) IR group, and (**c**,**f**) IR_PP group. * *p* < 0.05, ** *p* < 0.01, *** *p* < 0.001.

It is well known that metabolites play a crucial role in mediating metabolic and immune interactions between the microbiome and its host, thereby providing a fundamental perspective for understanding the complex dynamics of environmental exposure. Therefore, we constructed a joint network of the microbiome and metabolome based on Spearman correlation analysis. In the NC group, network analysis (Figure 6a) revealed multiple key interaction nodes. Choldienic acid, as a core metabolite, showed significant positive correlations with *unclassified_f_Lachnospiraceae* (ASV324, ASV326) and *Colidextribacter* (ASV188), collectively forming a functional module centered on bile acid metabolism. In contrast, triterpenoids showed a significant negative correlation with *unclassified_f_Oscillospiraceae* (ASV25). Heatmap analysis (Figure 6d) further indicated that Bacteroidota primarily correlated positively with amino acids and lipid metabolites, while Bacillota exhibited strong associations with triterpenoids and sterols. In the IR group (Figure 6b), the association between choldienic acid and Lachnospiraceae and *Colidextribacte* disappeared. Concurrently, *Marvinbryantia* (ASV29) and *unclassified_f_Oscillospiraceae* (ASV25) established new negative correlations with (+)-Quebrachitol, while *unclassified_f_Anaerotruncus* (ASV45) formed new positive correlations with multiple metabolites, including (-)-Wikstromol. The heatmap (Figure 6e) revealed weakened associations between Bacteroidota and amino acid/nitrogen metabolism, while Bacillota showed enhanced correlations with steroid/triterpenoid metabolites. In the IR_PP group, *P. petiolosa* intervention significantly restored some damage. The negative correlation (Figure 6c) between triterpenoids—present in the NC group but absent in the IR group—and *unclassified_f_Oscillospiraceae* (ASV25) reappeared. Furthermore, although the overall network has not fully recovered to the level of the NC group, it has clearly departed from the dysregulated state observed in the IR group and reassembled into a new ensemble of beneficial connections. Heatmap analysis (Figure 6f) further revealed that *P. petiolosa* enhanced the association between Bacillota and metabolites related to amino acids, lipids, and certain bile acids, while attenuating the excessively concentrated steroid-triterpenoid metabolic module present in the IR group.

## 3. Discussion

RIII is a severe complication of radiotherapy, characterized by a multifactorial pathophysiology that includes direct cellular damage, oxidative stress, inflammatory responses, and gut microbiota dysbiosis [18]. *P. petiolosa*, as a traditional Chinese medicinal herb, has been primarily used for heat-clearing and detoxification, diuresis [19], and reduction in swelling [20]. Clinically, it is commonly prescribed for urinary system disorders and inflammation-associated conditions [21]. However, systematic investigations into the use of *P. petiolosa* for RIII are lacking. This study systematically evaluated the protective effects of *P. petiolosa* against RIII, comprehensively examining its mechanisms at five levels—cellular, animal, metabolite, microbial, and combined analyses. The results indicate that *P. petiolosa* not only enhances antioxidant defenses and reduces inflammatory responses at the cellular level but also improves hematological parameters and tissue damage in animal models. More importantly, it can restore intestinal damage by reshaping dysbiotic microbiota and regulating disrupted metabolomes. The coordinated restoration of beneficial microorganisms and protective metabolites—such as short-chain fatty acids and bile acids—improved intestinal barrier integrity and inflammatory responses in irradiated mice. These findings provide experimental evidence supporting the therapeutic potential of *P. petiolosa* in RIII and theoretical support for developing novel natural radioprotective agents.

Ionizing radiation causes cellular damage through direct and indirect effects [22], further triggering a robust inflammatory cascade characterized by the massive release of multiple cellular inflammatory mediators [23]. Radiation significantly upregulates the mRNA expression of *TNF-α*, *IL-1β*, and *IL-6* in MODE-K cells, which is consistent with the literature reports indicating that these cytokines are key amplifiers of inflammatory signaling and exacerbators of tissue injury [24]. Simultaneously, ionizing radiation generates reactive oxygen species via water radiolysis [25], leading to disruption of intracellular redox homeostasis, a significant increase in MDA levels—a lipid peroxidation end product—and depletion of the primary intracellular antioxidants GSH and SOD. Following treatment with *P. petiolosa*, the expression of all three inflammatory factors was effectively suppressed, MDA levels decreased, and GSH and SOD levels were restored, indicating that *P. petiolosa* can simultaneously mitigate inflammatory amplification and oxidative damage while enhancing endogenous cellular antioxidant defense capabilities. *P. petiolosa* further exerted significant systemic protective effects within the body, not only by reducing the serum levels of TNF-α and IL-1β but also by decreasing MDA production while restoring GSH and SOD levels. Ionizing radiation also compromises hematopoiesis [26], producing leukopenia and lymphopenia—features of the hematopoietic subsyndrome of acute radiation syndrome. The observed differential recovery patterns in vivo, such as the rapid restoration of hematological parameters synchronized with delayed normalization of body weight, suggest that *P. petiolosa* preferentially protects radiation-sensitive proliferative tissues like the hematopoietic system and intestinal crypts. This may occur by promoting stem cell survival and differentiation [27], while systemic metabolic recovery follows a slower trajectory. Histological observations of restored intestinal architecture provide morphological validation for these mechanisms, demonstrating that reduced inflammation and oxidative damage directly translate into the preservation of the crypt–villus axis and barrier function, which are critical for defense against potential intestinal pathogens [28].

Another characteristic feature induced by ionizing radiation is gut microbiota dysbiosis [29]. Our findings align with this; irradiation reduced α-diversity and altered β-diversity structures, a recognized contributor to the pathogenesis of RIII [30]. The loss of diversity undermines ecosystem stability and functional redundancy, increasing susceptibility to invasion by opportunistic pathogens [31]. Intervention with *P. petiolosa* partially restored microbial diversity and structural stability, forming a crucial foundation for its protective effects.

At the taxonomic level, radiation-induced gut microbiota changes manifest as a decrease in the Bacteroidetes and Bacillota ratio and a significant expansion of Pseudomonadota [32,33]. The Bacteroidetes and Bacillota ratios and an “expansion” of Pseudomonadota are hallmark dysbiosis markers in inflammatory bowel disease and various metabolic disorders [34]. Pseudomonadota encompasses numerous opportunistic pathogens [35], such as the Enterobacteriaceae. The lipopolysaccharides, in their outer membranes, act as potent endotoxins that activate host inflammatory pathways and impair intestinal barrier function, thereby aggravating radiation injury. Intervention with *P. petiolosa* appeared to constrain Pseudomonadota overgrowth. Genus-level analysis provides deeper insight into the mechanism of action of *P. petiolosa*. We observed a significant increase in the abundance of *Ligilactobacillus*. *Ligilactobacillus* is a member of *Lactobacillus* [36]. It can inhibit pathogenic bacteria by secreting hydrogen peroxide [37] and modulate Th1/Th2 immune balance to alleviate allergic responses [38]. Its metabolites improve intestinal barrier function, and when combined with resveratrol, it synergistically alleviates colitis symptoms [39]. As “prebiotics” or selective antibacterial agents, *P. petiolosa* modulates the composition of gut microbiota. They can be metabolized by gut bacteria [40] to yield secondary metabolites with higher biological activity while preferentially supporting beneficial taxa and constraining opportunists. This directly or indirectly reshapes the intestinal microenvironment, thereby exerting its radiation-protective effects.

The functional consequences of microbial reshaping are clearly reflected in the fecal metabolome. Metabolomic profiling showed irradiation-associated perturbations in nucleotide, amino acid, and lipid metabolism [41], consistent with the DNA-repair defects and compromised membrane integrity characteristics of radiation injury [42]. Significant alterations in the arachidonic acid metabolic pathway, along with the upregulation of lipid oxidation products, such as 12(S)-HPETE, clearly indicate a radiation-triggered inflammatory cascade [43]. Concurrently, disruptions in primary bile acid biosynthesis and secretion pathways align with published reports indicating that intestinal injury and dysbiosis severely impair bile acid metabolism and signaling [44]. The key role of *P. petiolosa* is reflected in its regulation of lipid metabolism, particularly through its significant impact on glycerophospholipid metabolic pathways. Glycerophospholipids form the fundamental backbone of cell membranes and organelle membranes, playing a crucial role in maintaining the physical integrity and function of the intestinal epithelial barrier [45,46]. *P. petiolosa* regulation of this pathway, together with its effects on choline metabolism, collectively points to a core protective mechanism: promoting the repair and regeneration of damaged intestinal epithelial cell membranes, thereby restoring intestinal barrier function.

Integrated microbial–metabolite correlation analysis provides the most compelling evidence for the mechanism of action of *P. petiolosa*, unifying all our observations. Under healthy conditions, gut microbiota and host metabolites form a highly structured and functionally specialized cooperative network. The positive correlation between the *unclassified_f_Lachnospiraceae* and *Colidextribacter* with cholanic acid highlights the crucial role of short-chain fatty acid-producing microbiota in maintaining bile acid metabolic homeostasis [47], which is essential for gut health. Ionizing radiation disrupts existing symbiotic relationships and alters microbial abundance, representing a core pathway in the pathological process of RIII [48]. *P. petiolosa* appears to act as an ecosystem-level modulator, rewiring the disrupted microbe–metabolite network toward a near-restored steady state. A key line of evidence is the restoration of a hallmark association from the healthy network within the IR_PP group: the negative correlation between triterpenoids and *Oscillospiraceae*. Furthermore, beneficial bacteria such as *Bacteroides* and *Ligilactobacillus* emerged as new core nodes in the restored network, establishing positive correlations with potentially protective metabolites. This aligns with our earlier observations: partial recovery of microbiome diversity and a healthier network structure; pathways related to carbohydrate metabolism, phospholipid metabolism, and antioxidant defense were enhanced. Collectively, while these multi-omic patterns point to a tight mathematical coupling between microbial shifts and metabolic profiles during recovery, they represent statistical associations that await further functional validation to establish direct causality.

Notably, LC-MS profiling provided a plausible chemical basis for the protective effects of *Pyrrosia petiolosa*. The aqueous extract was enriched in polar and moderately polar constituents, particularly phenolic acids and flavonoids, including quinic acid, caffeic acid, chlorogenic acid, eriodictyol, and naringenin (Table S1). These compounds are mechanistically consistent with the phenotypic improvements observed in the present study, as previous work has linked chlorogenic acid [49] and flavonoid constituents [50] to the attenuation of oxidative epithelial injury, suppression of NF-κB-associated inflammatory signaling, activation of Nrf2-dependent antioxidant responses, and preservation of barrier integrity. In light of the reductions in *TNF-α*, *IL-1β*, *IL-6*, and MDA, together with the restoration of GSH and SOD, the protective effects of *P. petiolosa* are unlikely to be attributable to a single molecule, but are more plausibly explained by the coordinated actions of phenolic and flavonoid constituents. This compositional profile also aligns with the microbiota and metabolic reprogramming observed in the present study, supporting the idea that host protection, microbiota remodeling, and metabolic restructuring represent interconnected outcomes along a shared host–microbiota–metabolism axis.

The present study supports the potential of *P. petiolosa* as a promising natural radioprotective agent. Nevertheless, several limitations should be acknowledged.

First, the aqueous extract of *P. petiolosa* is a chemically complex mixture. Although the extraction method is consistent with traditional decoction techniques and preserves polar or moderately polar components such as phenolic acids and flavonoids, the observed protective effects cannot be attributed to a single compound. LC–MS results indicate that multiple components, including chlorogenic acid, caffeic acid, and pinobanksin, may act synergistically to produce biological effects. Therefore, future studies involving extract fractionation, validation of purified compounds, pharmacokinetic analysis, and target identification are needed to identify the primary active components and their mechanisms of action.

Second, the animal experiments still have design limitations. The specific dose of *P. petiolosa* extract was selected based on previous literature, but no dose-escalation experiments were conducted; therefore, the minimum effective dose, optimal dose, therapeutic window, and safety margin remain unclear. Furthermore, as with many models of RIII [51,52,53], this study used only male mice to minimize variability associated with the estrous cycle, which limits the generalizability of the findings to both sexes. The observation period was also limited to the acute phase following irradiation, and long-term survival rates, tissue integrity, and delayed damage were not evaluated. The absence of vehicle and positive controls further limits the assessment of nonspecific effects of administration and relative efficacy. Moreover, although histological analysis showed clear structural improvement of intestinal tissue after *P. petiolosa* treatment, specific markers of intestinal barrier integrity were not examined. Therefore, further testing—such as for ZO-1, claudin, tight junction protein-1, and MUC2—is needed to determine whether the observed morphological recovery is accompanied by the restoration of intestinal barrier function. The lack of vehicle and positive controls further limits the assessment of nonspecific administration effects and relative efficacy.

Third, integrated microbiome–metabolome analysis in this study is primarily based on correlation analysis; therefore, it can only reflect the association between changes in the gut microbiota and alterations in fecal metabolites rather than directly proving a causal relationship between the two regarding the protective effects of *P. petiolosa*. Furthermore, fecal metabolites may originate from host metabolism, gut microbial metabolism, diet, or the administered *P. petiolosa* extract and its metabolites. Therefore, the current results provide potential microbiome–metabolite clues rather than definitive evidence of mechanisms. Future studies should utilize methods such as fecal microbiota transplantation, antibiotic-mediated microbiota modulation, germ-free animal models, and targeted metabolite supplementation to validate the causal contribution of the gut microbiota and related metabolites to the protective effects of *P. petiolosa*.

## 4. Materials and Methods

### 4.1. Preparation of Aqueous Extract of P. petiolosa

*P. petiolosa* were collected in June 2016 from Mianxi Town, Wenchuan County, Aba Tibetan and Qiang Autonomous Prefecture, Sichuan Province. Identification was conducted by Associate Professor Bai Jie of the School of Life Sciences, Sichuan University, based on the characteristics of rhizome scales, leaves, and sporangia. Key features included ciliate margins on rhizome scales. The leaf underside bears only a single layer of stellate trichomes, with unipinnate, lanceolate branches exhibiting a length-to-width ratio of 3:1. Leaves are slightly dimorphic and leathery; they possess long petioles, typically 1–2 times the leaf blade length. The leaf blade is oblong, often 3–6 cm long, widest at the middle; the apex is rounded to obtuse, the base bilaterally symmetrical, cuneate, and extending downward. The leaf blade is often involute, covered with thick stellate hairs; lateral veins are inconspicuous; indusia are densely scattered on the leaf underside, spreading and coalescing at maturity. The type specimen (accession number SZ 162295) is deposited in the Herbarium of the Natural History Museum, Sichuan University, China.

One hundred grams of *P. petiolosa* was weighed and pulverized with a high-speed pulverizer. The powder was soaked in 1500 mL of ultrapure water for 30 min and then decocted. After boiling, it was cooked over a slow fire for 30 min, and the medicinal liquid was collected by filtering through a four-layer gauze. The above operation was repeated, and the two medicinal liquids were combined, cooked over a slow fire until boiling, and concentrated to 100 mL (final concentration 1.0 g/mL). The final aqueous extract was stored in a refrigerator at 4 °C for later use. The chemical composition of the aqueous extract of *P. petiolosa* was preliminarily characterized using LC–MS; the relevant methods and results are provided in the Supplementary Materials (Figure S1 and Table S1).

### 4.2. Cell Culture

The MODE-K cell was provided by Qingqi (Shanghai) Biotechnology Development Co., Ltd. (Shanghai, China). The mouse intestinal epithelial cells MODE-K, stored at the Key Laboratory of Biological Resources and Ecological Environment, Ministry of Education (Chengdu, China), were cultured in DMEM medium (Servicebio, Wuhan, China) supplemented with 10% fetal bovine serum (Servicebio) and maintained at 37 °C in a humidified incubator with 5% CO_2_ and 95% air.

### 4.3. Animals

Experiments were performed in 6–8-week-old male Balb/c mice with body weights of 20–25 g, which were obtained from the Animal Experiment Center of Sichuan University (Chengdu, China).

The animal production license number is SCXK (Chuan) 2025–0026. Animal care and all experimental procedures were conducted in accordance with the Animal Care Committee guidelines and approved by The General Hospital of Western Theater Command Ethics Committee (ethics approval number: 2025EC1-ky016; ethics approval date: 10 February 2025). Mice were housed under standard conditions, with food and water available at all times.

### 4.4. Irradiation Treatment

MODE-K cells (5 × 10^3^ cells/well) were seeded in 96-well plates and cultured in a DMEM medium containing 10% serum for 24 h. Cells were irradiated using the RS 2000 Pro biological irradiator (Rad Source Technologies, Buford, GA, USA) at a dose rate of 3.1 Gy/min. Doses of 3, 6, 9, 12, 15, and 18 Gy were administered to determine the optimal irradiation dose for the cells. MODE-K cells (5 × 10^5^ cells/well) were seeded in 6-well plates and cultured in a DMEM medium containing 10% serum for 24 h. After irradiating the cells with a total dose of 12 Gy at a rate of 3.1 Gy/min, the extract was added to the IR_PP group. The cells were divided into three groups: NC group: no irradiation, without the extract; IR group: radiation, without the extract; IR_PP group: radiation, with the extract. Cell samples were collected for testing 6 h and 24 h after irradiation.

Fifty-four mice were randomly divided into three equal groups, with two groups undergoing abdominal irradiation. Mice were anesthetized using 1.25% tribromoethanol (0.2–0.3 mL/10 g) and immobilized, with all areas except the abdomen shielded by lead plates. Following anesthesia, mice were irradiated using the irradiator at a dose rate of 1.14 Gy/min, delivering a total dose of 7.5 Gy. Mice were divided into three groups (*n* = 18): NC group: mice were gavaged orally with 200 μL of saline daily; IR group: mice were gavaged orally with 200 μL of saline daily and subjected to irradiation; IR_PP group: mice were gavaged orally with 200 μL of *P. petiolosa* solution (300 mg/mL) daily and subjected to irradiation. Daily observations and recordings were made of the mice’s survival status and weight changes. On days 1, 3, and 5, three mice were randomly selected for sample collection. On day 7, the remaining mice were fasted for 8 h before euthanasia, with blood and tissue samples collected. Fresh feces were collected prior to fasting for gut microbiota and metabolomics analyses.

### 4.5. Cytotoxicity Assay of P. petiolosa

The stock solution of *P. petiolosa* (1 g/mL) was stored at 4 °C and diluted with sterile water for use. A CCK-8 (Cell Counting Kit-8, Labgic Technology, Beijing, China) assay was used to examine the cytotoxicity of *P. petiolosa* and determine the optimal irradiation dose for cells. The concentrations of *P. petiolosa* were 0.01, 0.05, 0.1, 0.25, 0.5, 1, and 2 mg/mL. The time point of co-cultivation was 24 h.

### 4.6. Real-Time PCR

Total RNA of cells was extracted using an RNA extraction kit (Jianshi Biotech, Beijing, China). The total RNA was subjected to cDNA synthesis with PrimeScript^TM^ FAST RT reagent Kit with gDNA Eraser (Takara Biomedical Technology, Beijing, China). The qPCR was performed with TB Green^®^ Premix Ex TaqTM II FAST qPCR (Takara Biomedical Technology, Beijing, China). The primers used for PCR amplification were shown as follows: 5′-GAGAGTGTTTCCTCGTCCCGTAG-3′, 5′-CAACAATCTCCACTTTGCCACTG-3′ (*GAPDH*), 5′-GACTTCCATCCAGTTGCCTT-3′, 5′-ACAACTCTTTTCTCATTTCCACGA-3′ (*IL-6*), 5′-CCCACGTCGTAGCAAACCA-3′, 5′-ACAAGGTACAACCCATCGGC-3′ (*TNF-α*), 5′-TGCCACCTTTTGACAGTGATG-3′, 5′-AAGGTCCACGGGAAAGACAC-3′ (*IL-1β*). *GAPDH* was used as a normalizing control. The fold changes in mRNA were calculated with the 2^−ΔΔCt^ method.

### 4.7. Peripheral Blood Analysis

Blood was collected in pre-coded EDTA-containing vials on days 1 and 7 after irradiation. Blood was mixed gently on a rotary shaker until analysis on a hematology analyzer BC 2800Vet (Servicebio, Wuhan, China).

### 4.8. Antioxidant Assays

Mouse blood was centrifuged at 3000 r/min for 20 min at 4 °C, and the serum was collected for subsequent analysis. The serum and the cells were collected after irradiation for GSH, MDA, and SOD testing. The GSH was performed following the instructions on the Reduced Glutathione (GSH) Assay Kit. The MDA was performed following the instructions on the Malondialdehyde (MDA) Content Assay Kit. The SOD was performed following the instructions on the Superoxide Dismutase (SOD) assay kit (WST-1 method).

### 4.9. ELISA Assay

The serum was collected on days 1, 3, 5, and 7 after irradiation for ELISA testing. This ELISA was performed following the instructions on the ELISA Kit. Mouse ELISA kits of TNF-α and IL-1β were obtained from Jiangsu Meimian Industrial Co., Ltd. (Yancheng, China).

### 4.10. HE Staining and IHC Experiments

Mouse jejunum tissues were collected on days 1, 3, 5, and 7 after irradiation. Jejunum tissues were fixed in 4% paraformaldehyde for at least 24 h, processed using graded ethanol dehydration (50, 70, 95, and 100% ethanol), and embedded in paraffin wax. Paraffin blocks were sectioned at 4–5 μm thickness, stained with hematoxylin and eosin, and examined under a microscope to analyze histological changes.

For immunohistochemical staining, tissue sections collected on day 7 post-irradiation were subjected to antigen retrieval in EDTA buffer. Endogenous peroxidase activity was blocked by incubation with 3% hydrogen peroxide, after which the sections were washed with PBS. The sections were then blocked with 3% BSA for 1 h and incubated overnight at 4 °C in a humidified chamber with the primary antibody diluted in PBS at the indicated ratio. After washing with PBS, the sections were incubated with the appropriate secondary antibody for 50 min. Signals were developed using DAB, and the sections were subsequently counterstained with hematoxylin, dehydrated, and mounted. Primary antibody: rabbit anti-Ki67; secondary antibody: HRP-conjugated goat anti-rabbit IgG.

### 4.11. Gut Microbiota Analysis

The collected fecal samples were shipped to Shanghai Meggie Biomedical Technology Co., Ltd. (Shanghai, China) on dry ice for 16S ribosomal DNA sequencing and analyses, with both positive ion (POS) mode and negative ion (NEG) mode used. The amplification primers of the 16S rRNA gene were 338F and 806R.

### 4.12. Untargeted Metabolomics

Fecal samples were collected and stored at −80 °C. A 100 mg sample was added to a 2 mL centrifuge tube, and a 6 mm diameter grinding bead was added. A total of 800 μL of extraction solution was used for metabolite extraction. Samples were ground by the Wonbio-96c (Shanghai Wanbo Biotechnology Co., Ltd., Shanghai, China) frozen tissue grinder for 6 min (−10 °C, 50 Hz), followed by low-temperature ultrasonic extraction for 30 min (5 °C, 40 kHz). The samples were left at −20 °C for 30 min, centrifuged for 15 min (4 °C, 13,000 r/min), and the supernatant was transferred to the injection vial for LC-MS analysis.

The LC-MS analysis of the sample was conducted on a UHPLC-Orbitrap Exploris 240 system equipped with an ACQUITY HSS T3 column (Waters, Milford, MA, USA) at Majorbio Bio-Pharm Technology Co., Ltd. (Shanghai, China). The mobile phases consisted of 0.1% formic acid in water: acetonitrile (2:98, *v*/*v*) (solvent A) and 0.1% formic acid in acetonitrile (solvent B). The flow rate was 0.40 mL/min, and the column temperature was 40 °C. The injection volume was 5 μL. The MS conditions were as follows: source temperature at 400 °C; sheath gas flow rate at 40 arb; aux gas flow rate at 10 arb; ion-spray voltage floating (ISVF) at −2800V in negative mode and 3500 V in positive mode, respectively. Data acquisition was performed using the Data Dependent Acquisition mode. Detection was carried out over a mass range of 70–1050 *m*/*z*.

### 4.13. Statistics and Data Analyses

Data are expressed as mean ± SD. The statistical significance was analyzed with GraphPad Prism 10.1.2 software using one-way ANOVA followed by Tukey’s multiple comparisons test or a two-way ANOVA. Significance was set at *p* < 0.05, *p* < 0.01, *p* < 0.001, and *p* < 0.0001, denoted *, **, ***, and ****, respectively, in the figures. The omics data were analyzed through the free online platform of the Majorbio Cloud platform (https://www.majorbio.com/).

## 5. Conclusions

In summary, *P. petiolosa* attenuates inflammation and oxidative stress while promoting mucosal barrier repair at the cellular and animal levels. In parallel, it helps maintain intestinal homeostasis by reshaping gut microbial and rebalancing perturbed metabolic pathways. Multi-omics evidence reveals its extensive and multi-layered mechanisms of action, suggesting application potential across diverse individuals and complex environments. Consequently, *P. petiolosa* represents a promising candidate for the development of natural radioprotective strategies.

## Data Availability

This study did not involve any human subject research. Data from this study were not deposited in any external repository. Additional information supporting the research findings may be obtained upon reasonable request to the corresponding author.

## Supplementary Materials

The following supporting information can be downloaded at: https://www.mdpi.com/article/10.3390/ijms27125279/s1.
