# Supplementary material for: Metabolomic and Microbiome Profiling Reveals the Protective Mechanism of Pyrrosia petiolosa Against Radiation-Induced Intestinal Injury
Source: Int J Mol Sci. 2026 Jun 10;27(12):5279. doi: 10.3390/ijms27125279 (PMC13300518; doi:10.3390/ijms27125279)
Supplement: Supplementary file 1 [file ijms-27-05279-s001.zip › ijms-4288283-supplementary.pdf]

LC-MS compositional analysis of aqueous extract of *Pyrrosia petiolosa*

MS conditions: An ESI ion source was used, with the ESI capillary voltage set to +3.0 kV in positive ion mode and -2.5 kV in negative ion mode; ion source temperature was 120℃; heated capillary temperature was 450℃; desolvation gas flow rate was 800 L/h; cone voltage was 50 V; cone gas flow rate was 50 L/h; Mass scan range for both positive and negative ions: 50-1000 m/z; experimental data were processed using Waters UNIFI Portal software. Chromatographic conditions: Column: Intersustain C18 (4.6 mm × 250 mm, 5 μm); Wavelength: 265 nm; Mobile phase A: Acetonitrile solution; Mobile phase B: 0.1% formic acid aqueous solution (pH neutral); Flow rate: 1.0 mL/min; Injection volume: 10 μL; Column temperature: 35℃; Gradient elution program: 0 min-5% A, 15 min-20% A, 20 min-50% A, 25 min-90% A, 30 min-95% A, 35 min-95% A.

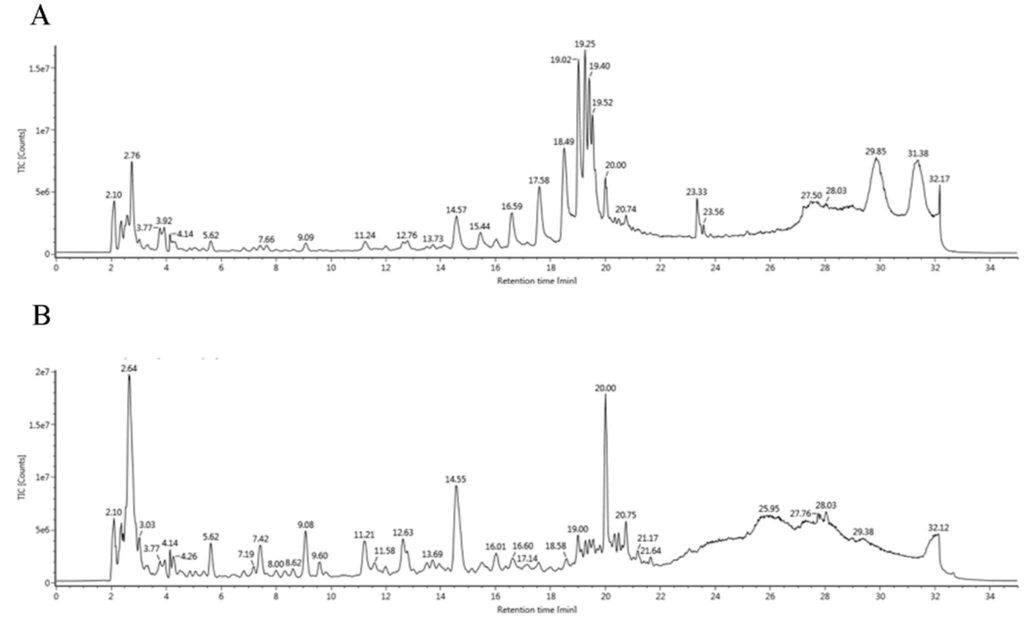

Supplementary Figure S1 Total ion chromatogram (TIC) of aqueous extract of *P. petiolosa*

Supplementary Table S1 UPLC/Q-TOF/MS analysis of aqueous extract of *P. petiolosa*

No.	t_R/min	Ion type	MS (m/z)	Molecular formula	Compound	i-FIT Confidence (%)
1	2.6762	$[M-H]^-$	191.05635	C7H12O6	Quinic acid	99.999840
2	3.0372	$[M-H]^-$	281.08773	C10H18O9	Xylobiose	100.000000
3	3.2987	$[M-H]^-$	337.07722	C12H18O11	Ascorbyl glucoside	99.959088
4	4.2349	$[M+Na]^+$	215.01577	C6H8O7	Citric acid	99.974880
5	6.8423	$[M-H]^-$	383.15542	C15H28O11	n-Propyl β-lactoside	98.667076
6	8.0812	$[M+H]^+$	224.12794	C12H17NO3	Cerulenin	100.000000
7	11.2450	$[M+H]^+$	163.03853	C9H6O3	Hydroxycoumarin	100.000000
8	11.6594	$[M-H]^-$	179.03473	C9H8O4	Caffeic acid	99.998899
9	14.6042	$[M+Na]^+$	377.08424	C16H18O9	Chlorogenic acid	99.941389
		$[M-H]^-$	353.08742	C16H18O9		99.994529
10	20.0074	$[M+H]^+$	289.07054	C15H12O6	Eriodictyol	99.989731
11	20.7450	$[M+H]^+$	273.0752	C15H12O5	Naringenin	98.948296
12	23.3527	$[M+H]^+$	318.30049	C18H39NO3	Phytosphingosine	100.000000
13	31.3673	$[M+H]^+$	338.34144	C22H43NO	Erucamide	100.000000
